# Kinetic and temporospatial parameters in male and female cats walking over a pressure sensing walkway

**DOI:** 10.1186/1746-6148-9-129

**Published:** 2013-06-27

**Authors:** Mirela R Verdugo, Sheila C Rahal, Felipe S Agostinho, Verônica M Govoni, Maria J Mamprim, Frederico OB Monteiro

**Affiliations:** 1 Department of Veterinary Surgery and Anesthesiology, School of Veterinary Medicine and Animal Science – Univ Estadual Paulista (UNESP), Botucatu, SP, Brazil; 2 Department of Animal Reproduction and Veterinary Radiology, School of Veterinary Medicine and Animal Science – Univ Estadual Paulista (UNESP), Botucatu, SP, Brazil; 3 Universidade Federal Rural da Amazônia, Instituto de Saúde e Produção Animal, Belém, do Pará, Brazil

## Abstract

**Background:**

Several factors may influence kinetic data measurements, including body conformation and body mass. In addition, gender differences in gait pattern have been observed in healthy humans. Therefore, the aim of this study was to compare the kinetic and temporospatial parameters in clinically healthy male and female cats using a pressure-sensitive walkway. Eighteen crossbreed adult cats were divided into two groups: G1 had ten male cats (nine neutered) aged from 1 to 4 years and body mass 3.1-6.8 kg; G2 had eight spayed female cats, aged from 1 to 6 years and body mass 3.3-4.75 kg. The data from the first five valid trials were collected for each cat. A trial was considered valid if the cat maintained a velocity between 0.54-0.74 m/s and acceleration from -0.20 to 0.20 m/s^2^. The peak vertical force (PVF), vertical impulse (VI), gait cycle time, stance time, swing time, stride length, and percentage body weight distribution among the four limbs were determined. In addition, the lengths of each forelimb and each hind limb were measured using a tape with the animal standing.

**Results:**

No significant differences were observed in each group in either the forelimbs or the hind limbs or between the left and right sides for any of the variables. For both groups, the PVF (%BW), the VI, and the percentage body weight distribution were higher at the forelimbs than the hind limbs. The stride length was larger for males; however, the other kinetic and temporospatial variables did not show any statistically significant differences between the groups. The lengths of the forelimbs and hind limbs were larger in the male cats. There was a significant moderate positive correlation between the stride length and the length of the limbs.

**Conclusions:**

In conclusion, the only difference observed between male and female cats was the stride length, and this was due to the greater body size of male cats. This difference did not affect other temporospatial or kinetics variables.

## Background

In small animals, instrumented gait analysis has generally been performed more frequently in dogs; kinetic and kinematic methods or a combination of both methods have been used [1-4]. Consequently, knowledge of the ground reaction forces and joint angles as well as the temporospatial parameters has provided a better understanding of gait biomechanics and characteristics and has allowed a better clinical application of the gait analysis [3,4], although the cost and complexity of these systems restricts gait analysis to laboratories [5]. Gait examination in cats has been accomplished most commonly by visual methods. The exam is performed in closed rooms or by evaluating videotape that is recorded in the home environment, as these animals seldom agree to walk on a leash and refuse to move freely in an unfamiliar environment [6]. However, objective analysis can be done in cooperative or trained cats [5,7-12].

Kinetic analysis provides information about the forces produced during the gait cycle; the ground reaction force and the joint reaction force are most studied [1,13,14]. The measurement of ground-reaction forces is important to predict, describe and assess gait disabilities and functional outcome [4,13,14]. In small animals the ground-reaction forces have been more frequently measured by force platform; however, the use of pressure sensing walkway has increased, as this system allows the recording of multiple foot strike data in a single passage and may be used with small breed dogs and cats [2-4,7-9,11,15-19]. Another advantage of pressure sensing walkway technology is that it allows to gather data from left and right limbs in one pass over the measuring area, resulting in easy calculation of (a)symmetry ratios and symmetry index [18,19]. In addition, pressure plate equipment is not restricted to high-tech gait laboratories, unlike 3D kinematics and force plate analysis [19,20].

There are differences between the absolute force values recorded by the force platforms and those collected by pressure sensing systems, which are generally lower [2,15,16,20,21]. Several reasons have been proposed to explain the differences such as calibration techniques, type of sensors, and sampling frequency [15,21]. Therefore, the results obtained by different systems are not interchangeable [21,22]. Peak vertical force (PVF) and vertical impulse (VI) are the most commonly parameters used to analyse normal and abnormal gait in small animals [2-4,12,23,24], and PVF is the only force measured by pressure sensing walkway [2,7-9,15,16,25]. In addition to ground reaction forces, temporal and distance parameters of gait such as stride length, stride time, stance time, percentage of stance, swing time, and velocity may also be evaluated [17,18,26].

Several factors may influence kinetic data measurements [1,4,23], including body conformation and body mass [20,23,27,28]. In addition, gender differences in gait pattern have been observed in healthy humans [29]. Therefore, the aim of this study was to compare the kinetic and temporospatial parameters in clinically healthy male and female cats. The hypothesis was that these parameters may have differences that are associated with sex or body conformation.

## Methods

This study was approved by the Ethics Committee of the School of Veterinary Medicine and Animal Science –UNESP Botucatu (n° 175/2011-CEUA). Permission to use the cats in the study was obtained from the owners.

Thirty clinically healthy cats recruited from client-owned or shelter-owned were used. The cats were judged to be healthy from complete physical, and orthopaedic examinations based on described by Voss and Steffen [6]. Although the cats did not have any signs of orthopaedic disease, digital radiographs only of the hip joints and stifle joints were obtained to complement clinical examination. One experienced radiologist reviewed the radiographs. Before the data collection, the cats were acclimatised to the room and stimulated to walk across the walkway for 1 hour or more. Each cat was weighed on the same electronic scale immediately before data collection.

### Data collection

The kinetic and temporospatial parameters of gait were measured with a 1951 mm × 447 mm pressure-sensitive walkway (Walkway High Resolution HRV4; Tekscan, South Boston, Massachusetts, USA) containing 33,408 pressure-sensitive cells. The sensors of the pressure-sensing walkway were equilibrated and calibrated using a phantom as reported previously [26] and according to the manufacturer’s specifications. During each trial, the cats were stimulated to walk in a straight line over the pressure-sensitive walkway; toys, food, and occasionally the presence of the owner were necessary to accomplish this task. In general, the stimulus was done with the materials positioned at the opposite end of the walkway. Boards measuring 2 X 0.8 m were placed on both sides of the walkway when deemed necessary.

For each animal, an average of 20 trials was obtained. The data from the first five valid trials were selected for each cat and analysed using designated software (Walkway 7.0; Tekscan, Boston, USA). A trial was considered valid if the cat walked at velocity within a previously determined range (0.54-0.74 m/s) and acceleration (-0.20 to 0.20 m/s^2^) parameters and if the 4 limbs had contacted with the surface of the walkway during each walk cycle.

For each limb, the gait cycle time (s), stance time (s) swing time (s) and stride length (m) were the determined temporospatial parameters. The stance time percentage was calculated as follows: (stance time/gait cycle time) × 100. The swing time percentage was calculated as follows: (swing time/gait cycle time) × 100. The stride corresponded to the distance between two consecutive ground contacts of the same limb.

The PVF and the VI were the kinetic parameters determined. The PVF and VI were normalised to the cat’s body weight and represented as a percentage of body weight, %BW and %BW*t, respectively. The percentage of body distribution among the four limbs was calculated as follows: (PVF of the limb/total PVF of the 4 limbs) × 100.

The duty factor in the hind limbs was calculated by dividing stance time by gait cycle time.

### Limb lengths

With the animal standing, the lengths of each forelimb and each hind limb were measured (cm), respectively, by the distance from the ground to the dorsal scapular rim and from the ground to the iliac crest using a generic plastic tape (metric).

### Symmetry ratio and maximum percentage of asymmetry

Since the side of asymmetry can vary, the symmetry ratio was calculated by dividing the highest value by the lowest value of each variable [30]. A mean (SD) of the 5 measurements of every animal was used. The maximum percentage of asymmetry was calculated by the formula: (Symmetry index – 1) × 100.

### Statistical method

To compare the temporospatial parameters and the kinetic data was used general linear model with sides (left and right) and sex (male and female) as fixed factors. An independent sample T-test was used to compare lengths of the forelimbs and hind limbs between groups. The values were expressed as the means ± standard deviation**,** and the coefficients of variation (CV) were calculated with the comparison data between the groups. Differences were considered significant at *P* < 0.05.

The symmetry index was expressed as mean ± standard deviation. The maximum percentage of asymmetry was determined by the upper percentage of the confidence interval calculated at 95% including all cats.

Pearson’s correlation coefficients (r) were used to evaluate the linear relationships between the limb lengths and the temporospatial parameters and kinetic parameters. The correlations were deemed significant at the 5% probability level.

## Results

All 30 animals were in the inclusion criteria of healthy animals. However, twelve cats were excluded because were not able to walk according to determined velocity, or in straight line without turn the head or without stop. The greatest difficulty was having the cat walk the course in a straight line at a constant velocity without any distractions. Only seven cats walked without use of boards.

From the 18 cats included in the study 16 were owned by clients, and two were shelter-owned. These cats were divided into two groups: G1 had 10 male crossbreed cats (of which nine were neutered) aged from 1 to 4 years and body mass 3.1-6.8 kg (mean 4.39 kg ± 1.14 SD); G2 had eight spayed female crossbreed cats aged from 1 to 6 years and body mass 3.3-4.75 kg (mean 3.82 kg ± 0.50 SD). The cats have been neutered/spayed from 11 months to 5 years. There were no body mass differences between the groups (*P* = 0.205).

No radiographic lesions were observed in the hip or stifle joints. The exception was a small meniscal mineralisation observed in both stifle joints in one cat of the G2, but without other clinical signs.

No significant differences were found between the kinetic data and the temporospatial parameters of the left and right forelimbs or the left or right hind limbs (Tables 1 and 2) in either group. In both groups, the PVF (%BW), VI, and the percentage of body weight distribution were higher at the forelimbs than the hind limbs (*P* < 0.001). The mean ± SD velocities were 0.63 ± 0.06 m/s and 0.61 ± 0.04 m/s in the male and female cats, respectively (*P* = 0.47).

**Table 1:** Comparison of the temporospatial parameters and the kinetic data between the right and left sides of the forelimbs of male and female cats

	**Male**		**Female**			
	**Right**	**Left**	**Right**	**Left**		
	**Mean ± SD**	**Mean ± SD**	**Mean ± SD**	**Mean ± SD**	***P *value**	**Power**
Stance Time (s)	0.48 ± 0.08	0.48 ± 0.07	0.46 ± 0.06	0.45 ± 0.07	0.850	0.054
Swing Time (s)	0.34 ± 0.04	0.34 ± 0.04	0.33 ± 0.03	0.33 ± 0.03	0.969	0.050
Gait cycle time (s)	0.80 ± 0.10	0.79 ± 0.10	0.76 ± 0.10	0.76 ± 0.09	0.962	0.050
Stride Length (m)	0.49 ± 0.02	0.49 ± 0.03	0.45 ± 0.01	0.45 ± 0.01	0.724	0.064
% of Stance	59.30 ± 3.61	59.72 ± 4.17	60.24 ± 2.78	59.12 ± 4.27	0.787	0.058
% of Swing	43.16 ± 2.61	42.41 ± 1.92	43.07 ± 4.11	43.62 ± 4.49	0.936	0.051
PVF (%BW)	54.54 ± 5.99	55.37 ± 4.99	55.15 ± 5.66	54.59 ± 7.83	0.742	0.062
VI (%BW*s)	18.08 ± 3.56	18.41 ± 2.78	18.36 ± 3.36	17.56 ± 4.33	0.428	0.122
% of Body Distribution	28.25 ± 1.39	28.75 ± 1.69	29.15 ± 0.99	28.31 ± 1.10	0.844	0.054

**Table 2:** Comparison of the temporospatial parameters and the kinetic data between the right- and left- side hind limbs of male and female cats

	**Male**		**Female**			
	**Right**	**Left**	**Right**	**Left**		
	**Mean ± SD**	**Mean ± SD**	**Mean ± SD**	**Mean ± SD**	***P *value**	**Power**
Stance Time (s)	0.46 ± 0.07	0.46 ± 0.09	0.45 ± 0.06	0.44 ± 0.06	0.859	0.053
Swing Time (s)	0.39 ± 0.05	0.41 ± 0.08	0.38 ± 0.04	0.40 ± 0.05	0.357	0.148
Gait cycle time (s)	0.83 ± 0.11	0.85 ± 0.15	0.81 ± 0.09	0.82 ± 0.08	0.778	0.059
Stride Length (m)	0.49 ± 0.02	0.49 ± 0.02	0.45 ± 0.01	0.44 ± 0.02	0.711	0.065
% of Stance	55.26 ± 3.85	54.47 ± 2.88	55.67 ± 4.98	53.38 ± 5.66	0.301	0.175
% of Swing	47.22 ± 3.39	48.25 ± 1.95	47.83 ± 6.66	49.59 ± 7.17	0.415	0.126
PVF (%BW)	42.05 ± 7.03	41.28 ± 6.19	40.97 ± 5.30	40.21 ± 5.45	0.711	0.065
VI (%BW*s)	13.36 ± 2.63	13.10 ± 2.60	13.38 ± 3.04	12.77 ± 3.21	0.360	0.147
% of Body Distribution	21.67 ± 1.56	21.31 ± 1.42	21.28 ± 1.00	20.86 ± 0.71	0.656	0.072

The stride length was larger for male cats; however, the other kinetic and temporospatial variables did not show any significant difference between the groups (Table 3).

**Table 3:** Comparison of the temporospatial parameters and the kinetic data between the forelimbs and hind limbs of male and female cats

	**Forelimb**						**Hind limb**					
	**Male**		**Female**				**Male**		**Female**			
	**Mean ± SD**	**CV**	**Mean ± SD**	**CV**	***P *value**	**Power**	**Mean ± SD**	**CV**	**Mean ± SD**	**CV**	***P *value**	**Power**
Stance Time (s)	0.48 ± 0.07	14.68	0.46 ± 0.07	15.32	0.453	0.114	0.46 ± 0.08	17.28	0.44 ± 0.06	13.19	0.491	0.104
Swing Time (s)	0.34 ± 0.04	11.70	0.33 ± 0.03	9.12	0.231	0.220	0.40 ± 0.06	14.96	0.39 ± 0.04	10.13	0.770	0.059
Stride Time (s)	0.80 ± 0.09	11.25	0.76 ± 0.10	13.07	0.319	0.166	0.84 ± 0.13	15.42	0.82 ± 0.08	9.80	0.532	0.094
Stride Length (m)	0.49 ± 0.02a	4.08	0.45 ± 0.02b	4.45	<0.001	0.996	0.49 ± 0.02a	4.08	0.44 ± 0.02b	4.49	<0.001	1.000
% of Stance	59.51 ± 3.08	5.18	59.68 ± 3.53	5.91	0.896	0.052	54.87 ± 3.34	6.09	54.53 ± 5.28	9.68	0.818	0.056
% of Swing	42.78 ± 2.26	5.28	43.35 ± 4.17	9.62	0.619	0.078	47.74 ± 2.75	5.76	48.71 ± 6.75	13.86	0.569	0.086
PVF (%BW)	54.95 ± 5.38	9.79	55.75 ± 7.66	13.74	0.724	0.064	41.66 ± 6.46	15.51	40.59 ± 5.21	12.83	0.603	0.080
VI (%BW*s)	18.24 ± 3.11	17.05	17.96 ± 3.77	20.99	0.355	0.149	13.23 ± 2.55	19.27	13.07 ± 3.04	23.26	0.329	0.161
% of Body Distribution	28.50 ± 1.53	5.37	28.92 ± 1.13	3.91	0.809	0.056	21.49 ± 1.47	6.84	21.07 ± 0.87	4.13	0.872	0.053

The values that are followed by different letters along each horizontal row are significantly different.

Significant differences were found between the forelimbs and hind limbs for both G1 and G2 in the swing time (*P* = 0.001, *P* < 0.001), swing percentage (*P* < 0.001, *P* = 0.011), stance percentage (*P* < 0.001, *P* = 0.002), PVF (*P* < 0.001, *P* < 0.001), VI (*P* < 0.001, *P* < 0.001), and body distribution percentage (*P* < 0.001, *P* < 0.001).

The symmetry rate and the maximum percentage of asymmetry of the kinetic and temporospatial variables are described in Table 4. The hind limb duty factors had a mean of 0.55 for the male cats and 0.54 for the female cats. There were differences in the lengths of the forelimbs and the hind limbs, which were both larger in the male cats (Table 5). There was a significant moderate positive correlation between the stride length and the length of the limbs (Table 6).

**Table 4:** Symmetry ratio between the right and left sides and the maximum percentage of asymmetry of the temporospatial parameters and kinetic data of the forelimbs and hind limbs of all cats

**Variables**	**Symmetry ratio left/right side**		**Maximum percentage of asymmetry**	
	**Forelimb**	**Hind limb**	**Forelimb**	**Hind limb**
Stance Time (s)	1.05 ± 0.05	1.05 ± 0.04	8.5%	7.6%
Swing Time (s)	1.06 ± 0.06	1.08 ± 0.06	6.9%	11.9%
Gait cycle time (s)	1.04 ± 0.03	1.03 ± 0.03	6.1%	5.3%
Stride Length (m)	1.01 ± 0.01	1.01 ± 0.01	1.8%	2.4%
% of Stance	1.05 ± 0.05	1.05 ± 0.04	7.4%	7.7%
% of Swing	1.05 ± 0.04	1.07 ± 0.05	7.6%	10.3%
PVF (%BW)	1.05 ± 0.02	1.04 ± 0.02	6.2%	5.5%
VI (%BW*s)	1.09 ± 0.07	1.06 ± 0.05	12.8%	9.2%
% of Body Distribution	1.05 ± 0.02	1.04 ± 0.02	6.2%	5.5%

**Table 5:** Lengths of the forelimbs and hind limbs (cm) of male and female cats

	**Male**	**Female**	
	**Mean ± SD**	**Mean ± SD**	***P value***
Forelimbs	24.94 ± 1.94^**a**^	22.50 ± 0.83^**b**^	0.012
Hind limbs	25.61 ± 2.07^**a**^	23.33 ± 0.5^**b**^	0.024

The values that are followed by different letters along each horizontal row are significantly different.

**Table 6:** Pearson’s correlation coefficient between the temporospatial and kinetic values and lengths of the forelimbs and the hind limbs (cm) of male and female cats

**Variables**	**Forelimb**	**Hind limb**
Stance Time (s)	0.37	0.25
Swing Time (s)	0.30	0.28
Gait cycle time (s)	0.37	0.31
Stride Length (m)	0.68*	0.57*
% of Stance	0.21	−0.006
% of Swing	−0.25	−0.11
PVF (%BW)	−0.18	0.15
VI (%BW*s)	−0.43	0.30
% of Body Distribution	0.23	0.27

*Significant statistical correlation.

## Discussion

The ground reaction force measurements may be affected by several factors; therefore, velocity conditions must be controlled and a consistent gait pattern is necessary [1,23]. Cat behavior is challenging when performing gait analysis [6,11]. In the present study, only 18 of 30 cats could be used in the gait analysis, despite several types of stimuli. In another study, 15 of 23 cats proved satisfactory for gait analysis [9].

In the present study, the gait velocity varied from 0.54 to 0.74 m/s with no difference between the groups. This velocity was based on walking, as the hind limb duty factors were 0.55 and 0.54 for male and female cats, respectively. When the duty factor or the fraction of cycle time that the limb is on the ground is > 0.5, the gait is considered walking for quadrupeds; < 0.5 is suggestive of running or trotting [31].

Other studies that have used healthy cats walking on a pressure-sensitive walkway have reported velocities from 0.37 to 0.85 m/s (0.6 m/s mean) [9] or mean values of 0.69 ± 0.029 m/s [9] and 0.67 ± 0.22 m/s [11]. The velocity influences the ground reaction forces and the stance time [1,23]. If the range is too large, it is impossible to conclude whether the differences are related to abnormal gait or velocity variations [23]. In cats, the maximum variation of the velocity is undefined.

The stance time and stance time percentage were greater than the swing time and swing time percentage for both the forelimbs and the hind limbs, with no difference between the males and the females. This is consistent with walking when the stance phase is longer than the swing phase and is confirmed by the duty factor. The same result was observed in dogs walking on a pressure-sensitive walkway [18]. In addition, the stance duration decreases with increased velocity in a manner that is similar to the cycle duration in quadrupeds within a gait [31].

However, the stance time and gait cycle time were statistically similar between the forelimbs and the hind limbs for both groups, although the swing time was longer in the hind limbs. This difference influenced the percentages, i.e., the stance percentage was higher and the swing percentage was lower in the forelimbs. In addition, one study suggested that based on the front to hind limb ratio (1.07 ± 0.04), cats use their forelimbs slightly longer than their hind limbs during the stance phase [11].

The stride length for both the forelimbs and the hind limbs was longer in the male cats and showed a significant correlation to limb length. Thus, the difference is related to the body size of the male cat and not sex-related. In addition, there was no body mass difference between the groups. As an indicator of body size, the limb length influences the stride length and may influence other variables, such as stance time [28,32]. For small dogs walking on a pressure sensing walkway, most of the temporospatial and kinetic variables were significantly smaller than those of large dogs, and the small dogs had more frequent paw strikes and a shorter gait cycle [18]. The variability could be diminished using dynamic similarity. However, in a study was observed that hind limb PVF in ponies was slightly higher than in horses even though the ponies moved at the same relative velocity [20].

Generally, the vertical force is the maximum force that acts on the body [33]. For both male and female cats, the PVF and VI were higher in the forelimbs than the hind limbs. This finding was similar to previously reported data of healthy cats walking on a pressure sensing walkway [7,9,11] or force plate [33]. However, the mean values and proportions differed between the studies and may be associated with the methodologies used for pressure sensing walkway calibration [9] and the system types. Romans et al. [7] cited that differences between the forelimbs and the hind limb ground reaction forces could be less in cats than in dogs, as the mean PVF (%BW) obtained was 56.41 for the forelimbs and 50.22 for the hind limbs in healthy cats walking on a pressure sensing walkway. In our study, however, the mean PVF (%BW) values for the forelimbs were 54.95 ± 5.38 and 55.75 ± 7.66 in the male and female cats, respectively, and 41.66 ± 6.46 and 40.59 ± 5.21 for the hind limbs in the male and female cats, respectively. Additionally, in a study of quadruped dynamics, three-fifths of the total vertical force in walking cats was distributed on the forelimbs, and this distribution was associated with a centre-of-gravity location nearer the forelimbs and an impulse produced at the forelimbs [34].

Symmetry changes have been associated with lameness [35,36]; however, the determination of the rate considered normal and that considered abnormal must be determined, as there is no absolute symmetry, which was shown in an analysis of the inverse dynamics gait in dogs [37]. In the present study, the average symmetry rates ranged from 1.01 to 1.09 (asymmetry ranging from 1 to 9%) which reinforces the importance of bilateral evaluation in gait analysis. Variables such as swing time, swing percentage and impulse showed a maximum percentage of asymmetry greater than 10%, suggesting that these parameters should be interpreted with caution in the detection of asymmetries. Furthermore, the stride length, gait cycle time, the PVF and the body distribution percentage showed the highest degree of symmetry and the lowest values for maximum percentage of asymmetry, indicating that these variables seem more appropriate for detecting gait changes.

One of the limitations of the present study was that most tests had powers in the range of 0.1 and a power level of 0.8 is usually necessary to feel that type 2 errors (false negative) are unlikely. Thus, further researches using a larger population of cats are justified.

## Conclusions

The only difference observed between male and female cat was the stride length, which was caused by the larger body size of male cats. This difference did not affect other temporospatial or kinetic variables.

## Competing interests

The authors have declared that no competing interests exist.

## Authors’ contributions

MRV, SCR and FSA conceived and designed the study; VMG helped collected the data; MJM performed and interpreted radiology examinations and FOBM helped draft the manuscript; all authors read, contributed to and approved the final manuscript.
